# 1164. Antibacterial Utilization for Febrile Illnesses and Laboratory-Confirmed Bloodstream Infections in Northern Tanzania

**DOI:** 10.1093/ofid/ofac492.1001

**Published:** 2022-12-15

**Authors:** Ganga S Moorthy, Deng Madut, Kajiru G Kilonzo, Bingileki Lwezaula, Ronald Mbwasi, Blandina Mmbaga, James Ngocho, Wilbrod Saganda, Clinical Officer, John P Bonnewell, Manuela Carugati, Joseph R Egger, Julian Hertz, Michael J Maze, Venance Maro, John A Crump, Matthew Rubach

**Affiliations:** Duke University Medical Center, Durham, North Carolina; Duke University, Durham, North Carolina; Kilimanjaro Christian Medial University College, moshi, Kilimanjaro, Tanzania; Mawenzi Regional Referral Hospital, Moshi, Kilimanjaro, Tanzania; Kilimanjaro Christian Medical Centre, Moshi, Kilimanjaro, Tanzania; Kilimanjaro Christian Medical Centre, Tumaini University, Moshi, Kilimanjaro, Tanzania; Kilimanjaro Christian Medical Centre, Moshi, Kilimanjaro, Tanzania; Mawenzi Regional Referral Hospital, Moshi, Kilimanjaro, Tanzania; Roswell Park Comprehensive Cancer Center, Buffalo, New York; Duke University, Durham, North Carolina; Duke University, Durham, North Carolina; Duke University, Durham, North Carolina; University of Otago, Christchurch, Canterbury, New Zealand; Kilimanjaro Christian Medical Centre, Tumaini University, Moshi, Kilimanjaro, Tanzania; University of Otago, Christchurch, Canterbury, New Zealand; Duke University, Durham, North Carolina

## Abstract

**Background:**

Antimicrobial resistance is an important cause of morbidity and mortality globally; low- and middle-income countries (LMICs) face an especially high burden. Ineffective antimicrobial prescriptions and use of broad-spectrum agents contribute to resistance. We describe antibacterial prescribing patterns in patients with febrile illnesses and bloodstream infections (BSI) in northern Tanzania.

**Methods:**

We compared data from two hospital-based prospective cohort studies, cohort 1 (2011-2014) and cohort 2 (2016-2019), both enrolled febrile pediatric and adult inpatients in Moshi, Tanzania. Aerobic blood culture was obtained in all patients and standard methods were used to determine isolate antimicrobial susceptibility. To describe management of febrile illness, we analyzed antibacterial drug prescription prior to and after enrollment. Broad-spectrum antibacterials were categorized using published frameworks. Treatments were categorized as discordant if a blood culture isolate was not susceptible to the patient’s antibacterial regimen. We performed descriptive statistics and logistic regression to understand predictors of receiving an antibacterial.

**Results:**

In total, 2,176 febrile inpatients were enrolled. Antibacterials were administered to 430 (42.0%) and 501 (45.1%) patients prior to enrollment, and 930 (89.1%) and 1,060 (93.6%) during admission in cohorts 1 and 2, respectively. Infancy and duration of fever were associated with higher antibacterial prescribing prior to enrollment (Table 2). Broad-spectrum antibacterials were administered to 548 (52.5%) in cohort 1 and 682 (60.2%) in cohort 2. Laboratory-confirmed bacteremia was detected in 87 (4%) patients. Susceptibility results were available in 73 patients; of these, 41 (56.2%) received discordant antibacterials.

**Figure:**
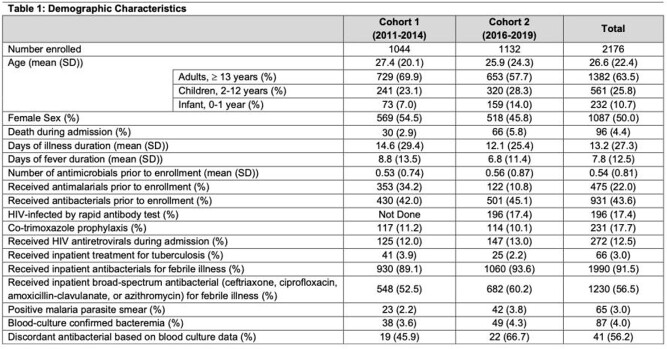

**Figure:**
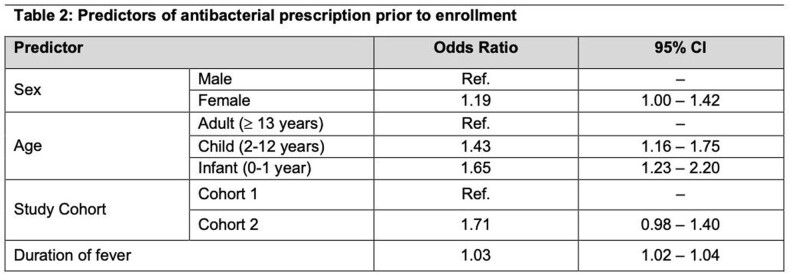

**Figure:**
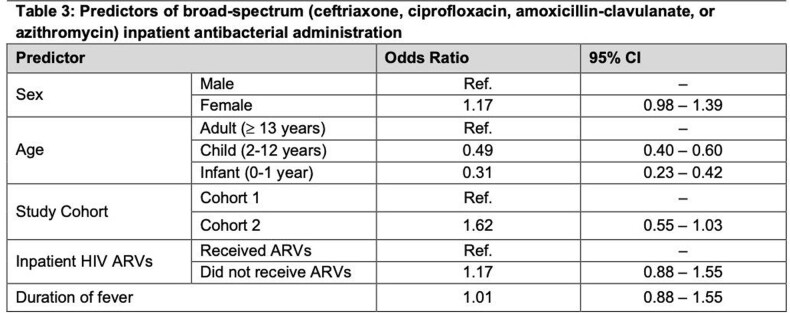

**Conclusion:**

Antibacterials were commonly administered for febrile illness in outpatient and inpatient settings; over half of patients received broad-spectrum antibacterials. A high proportion of patients with culture-positive BSI were treated with ineffective antimicrobials. Improved laboratory diagnostics for febrile illness, antimicrobial stewardship interventions, context-specific clinical guidelines, and provider education may improve prescribing practices.

**Disclosures:**

**Julian Hertz, MD**, Roche Diagnostics: Grant/Research Support.

